# Examining the impact of gestational diabetes genetic susceptibility variants on maternal glucose levels during and post pregnancy

**DOI:** 10.1136/bmjdrc-2025-005382

**Published:** 2025-12-03

**Authors:** Aminata H Cissé, Alan Kuang, Catherine Allard, Justiina Ronkainen, Robin N Beaumont, Sylvain Sebert, Denise M Scholtens, Andrew T Hattersley, Marja Vääräsmäki, Eero Kajantie, Luigi Bouchard, Patrice Perron, Elina Keikkala, Marie-France Hivert, William L Lowe Jr, Alice E Hughes, Rachel M Freathy

**Affiliations:** 1 Department of Clinical and Biomedical Sciences, Faculty of Health and Life Sciences, University of Exeter, Exeter, UK; 2 Department of Preventive Medicine, Northwestern University Feinberg School of Medicine, Northwestern University, Evanston, Illinois, USA; 3 Centre de Recherche du Centre Hospitalier Universitaire de Sherbrooke, Sherbrooke, Quebec, Canada; 4 Research Unit of Population Health, University of Oulu, Oulu, Finland; 5 Research Unit of Clinical Medicine, Medical Research Center Oulu, Oulu University Hospital and University of Oulu, Oulu, Finland; 6 Welfare Epidemiology and Monitoring Unit, Department of Public Health, Finnish Institute for Health and Welfare, Helsinki, Finland; 7 University of Helsinki and Helsinki University Hospital, Children’s Hospital, Helsinki, Finland; 8 Norwegian University of Science and Technology, Department of Clinical and Molecular Medicine, Trondheim, Norway; 9 Biochemistry and Functional Genomics Department, Faculty of Medicine and Health Sciences, Université de Sherbrooke, Sherbrooke, Quebec, Canada; 10 Department of Medicine, Faculty of Medicine and Health Sciences, Université de Sherbrooke, Sherbrooke, QC, Canada; 11 Department of Population Medicine, Harvard Pilgrim Health Care Institute, Harvard Medical School, Boston, MA, USA; 12 Department of Medicine, Massachusetts General Hospital, Boston, MA, USA; 13 Department of Medicine, Northwestern University Feinberg School of Medicine, Northwestern University, Evanston, Illinois, USA

**Keywords:** Diabetes, Gestational, Genetics, Cohort Studies, Meta-Analysis

## Abstract

**Aim:**

Genetic variants associated with gestational diabetes mellitus (GDM, n=14 SNPs) were recently classified into two groups: type 2 diabetes predominant effects (Class-T, three SNPs) and GDM-predominant effects (Class-G, eight SNPs; three SNPs unclassified). We aimed to compare the effects of GDM-associated variants on glucose levels (fasting glucose and 2-hour post-OGTT) measured during versus post pregnancy.

**Research design and Methods:**

We calculated genetic scores (GS) by class (T_GS and G_GS) and overall (All_GS) in 10 225 pregnant women and 4763 women post pregnancy (mean 10.5 years post pregnancy) from eight datasets representing four ancestrally-diverse cohorts: Exeter Family Study of Childhood Health, Genetics of Glucose Regulation in Gestation and Growth, Hyperglycaemia and Adverse Pregnancy Outcome, and Finnish Gestational Diabetes. We used linear regression models adjusted for ancestry principal components to investigate associations between standardized GS and glucose levels during or post pregnancy. Analyses were performed separately in each dataset and then combined using inverse-variance weighted random-effects meta-analyses.

**Results:**

All_GS was associated with fasting glucose both during and post pregnancy (β (95% CI), in mmol/L per 1 SD higher GS=0.06 (0.04 to 0.08) during vs 0.06 (0.04 to 0.07) post pregnancy). All_GS was also associated with 2-hour post-OGTT glucose levels during pregnancy but not after (0.10 (0.04 to 0.15) during vs 0.01 (−0.04 to 0.07) post pregnancy). Both G_GS and T_GS showed consistent associations with fasting glucose during and post pregnancy (0.06 (0.04 to 0.08) during and 0.05 (0.03 to 0.07) post pregnancy for G_GS; 0.02 (0.01 to 0.02) during and 0.02 (−0.001; 0.05) post pregnancy for T_GS). G_GS showed weak evidence of association with 2-hour glucose levels during pregnancy (0.06 (−0.002 to 0.11)) and no association with 2-hour glucose levels post pregnancy (−0.03 (−0.08 to 0.03)). However, T_GS was associated with 2-hour glucose during pregnancy and post pregnancy (0.10 (0.04 to 0.16) and 0.06 (0.01 to 0.12)).

**Conclusion:**

Consistent associations with fasting glucose levels during and after pregnancy may suggest that biological pathways underlying GDM genetic susceptibility to fasting hyperglycemia are not pregnancy specific. However, the results for All_GS and 2-hour glucose provide evidence that some genetic associations with postprandial glucose may be stronger in pregnancy and should be followed up in larger samples.

**WHAT IS ALREADY KNOWN ON THIS TOPIC**

- Genetic studies have identified variants that are associated with both gestational diabetes mellitus (GDM) and type 2 diabetes (T2D), pointing to a shared genetic background.
- GDM-associated variants have been grouped into two classes: those more strongly associated with GDM than T2D (Class-G), and those more strongly associated with T2D than GDM (Class-T).
- Class-G variants have been proposed to influence hyperglycemia predominantly during pregnancy, but this has not been tested.

**WHAT THIS STUDY ADDS**

- Genetic scores for GDM (combining class G, class T, or all variants) showed consistent associations with fasting glucose levels during and after pregnancy.
- Analyses of the full genetic score and class G score suggested that some GDM-associated variants were more strongly associated with 2-hour glucose levels during than after pregnancy, but larger samples are required to confirm this.

**HOW THIS STUDY MIGHT AFFECT RESEARCH, PRACTICE, OR POLICY**

- An improved understanding of how different GDM-associated variants influence glycemia inside and outside of pregnancy will inform our understanding of which women are more at risk of disease and how their glycemia relates both to pregnancy outcomes and to their future risk of T2D, enabling better targeting of care.

## Introduction

 Gestational diabetes mellitus (GDM), defined as glucose intolerance occurring or first observed during pregnancy, presents significant health risks for both mother and fetus.^1^ Offspring of mothers who have had GDM are more likely to have higher birth weight and to develop obesity and metabolic disorders at an earlier age.^2 3^ In addition, GDM is a strong risk factor for developing type 2 diabetes in later life.^4 5^ Up to 50% of women who have had GDM develop pre-diabetes or type 2 diabetes within 10–15 years post partum.^6^,^8^

Genome-wide association studies (GWAS) have made progress in identifying genetic loci underlying variation in GDM risk. The GENetics of Diabetes In Pregnancy Consortium performed a multi-ancestry meta-analysis of GWAS including 5485 women with GDM and 347 856 controls, identifying five loci robustly associated with GDM.^9^ Among these, four loci had previously been reported for type 2 diabetes, suggesting shared genetic pathways between the two conditions. Similarly, a study conducted in China on more than 30 000 pregnant women identified four loci associated with GDM, among which *MTNR1B* exhibited the strongest association with GDM while having a more modest effect on type 2 diabetes risk.^10^ Kwak *et al* also revealed that variants in *CDKAL1* and *MTNR1B* exhibited a stronger association with GDM than with type 2 diabetes in a two-stage genome-wide association analysis conducted among Korean women.^11^ The largest GWAS of GDM to date, conducted by Elliott *et al*, included 12 332 women with GDM and 131 109 controls from Finland. This study assessed the shared genetic etiology between GDM and type 2 diabetes.^12^ They identified 13 loci for GDM, many of which also influence type 2 diabetes risk, and classified the loci into two categories. In one category, the variants had stronger effects on type 2 diabetes than GDM (Class-T), and in the other (Class-G), the variants had stronger effects on GDM than type 2 diabetes. A possible inference from these findings was that Class-G loci related to mechanisms of glucose regulation specific to pregnancy. However, several of these loci were already known to influence other commonly measured glycemic traits in non-pregnant individuals without diabetes, and it is not known if their effects are altered by pregnancy status.^11 13^

To investigate the extent to which genetic susceptibility to GDM highlights glucose regulation mechanisms that are disrupted specifically during pregnancy, we aimed to compare the effects of groups of GDM-associated variants on fasting and 2-hour post-OGTT glucose levels measured during versus after pregnancy, across women of diverse ancestries. We performed a meta-analysis of eight datasets representing four cohorts: EFSOCH, Gen3G, HAPO, and FinnGeDi. We hypothesized that genetic scores (GSs) based on Class-G variants would show stronger associations with glucose levels during pregnancy than after pregnancy.

## Materials and methods

### Study descriptions

We analyzed data from 10 225 pregnant women and 4763 women post pregnancy across eight datasets representing four cohorts. The datasets included the Exeter Family Study of Childhood Health (EFSOCH), the Genetics of Glucose Regulation in Gestation and Growth (Gen3G) study, the Hyperglycaemia and Adverse Pregnancy Outcome (HAPO) study divided into five different ancestry groups based on genetic similarity (HAPO-European, HAPO-Afro-Caribbean, HAPO-East-Asian, HAPO-South-Asian and HAPO-Mexican-American), and the Finnish Gestational Diabetes (FinnGeDi) study.

The inclusion criteria required participants to have at least one glucose measurement during pregnancy and/or at least one glucose measurement post pregnancy, along with available genome-wide genotype data. Women with pre-existing type 1 or type 2 diabetes were excluded from all analyses. Women with glucose levels indicative of GDM, measured prior to any therapeutic intervention, were included in analyses regardless of subsequent treatment in most cohorts (see cohort specific details in online supplemental materials).

Details of genotyping and maternal glucose phenotype measurements for each cohort are provided in more detail in the online supplemental materials.

### Generating GSs

We used summary statistics from the GWAS of GDM published by Elliott *et al*^12^ to calculate the GSs, based on their classification. The overall GS (All_GS) included all identified variants (14 SNPs including three unclassified SNPs), while T_GS encompassed variants with stronger effects on type 2 diabetes than GDM (3 SNPs), and G_GS encompassed variants with stronger effects on GDM than type 2 diabetes (8 SNPs). A GS for an individual is equal to the sum of the genotype dosages for each GDM risk allele of each SNP, weighted by the log OR for GDM based on the GWAS summary statistics.^12^


$$GRSi=\;\sum_{i=0}^{N}\left(Gi\;\times \;\beta i\right)$$

Where *N* is the total number of variants included in the score, *G_i_* is the dosage of risk alleles for SNP (a number between 0 and 2 based on the individual’s genotype) and* β_i_* is effect size (log OR for GDM).

Each score was standardized within each cohort and ancestry group to a mean of 0 and SD of 1 for the regression analyses. A small number of SNPs were missing in different cohorts (online supplemental table S1).

### Statistical analysis

Basic descriptive maternal characteristics were summarized for each dataset during and after pregnancy.

We used linear regression to test the associations between the standardized GSs and glucose levels (fasting or 2-hour glucose levels, in mmol/L) during and after pregnancy, in each dataset separately. The main model was adjusted for the first five principal components of ancestry in all cohorts, except Gen3G, which included the first four principal components, and the variables linked to the specificities of each cohort, such as genotyping batches, center, etc. We referred to the ‘adjusted model’ as the model with additional adjustment for maternal age at the time of glucose measurement. Then, we performed inverse-variance weighted random-effects meta-analyses to combine the results from the eight datasets.

As a sensitivity analysis, we repeated the analyses including only women who had measurements both before and after pregnancy to test whether there was any difference in results when we compared the same women during and after pregnancy. To further explore whether the associations between GSs and glucose traits differed between pregnancy and post pregnancy, we fitted mixed-effects linear models within each cohort with a random intercept for participant ID to account for repeated measures, using the samples of women with both measurement during and after pregnancy. The models included the GS, pregnancy status (reference=pregnancy), and their interaction, along with covariates previously used in the main analyses (adjusted for principal components and cohort-specific covariates). Cohort-specific estimates of the interaction term and their SEs were then combined using random-effects meta-analysis. We also conducted a leave-one-out meta-analysis to see if a given cohort is particularly different from the others.

In addition, we investigated two SNPs known to have particularly strong associations with type 2 diabetes and fasting glucose, respectively: the *TCF7L2* (rs34872471) SNP from Class-T and *MTNR1B* (rs10830963) SNP from Class-G have strong associations in non-pregnant individuals compared with other glycemic trait SNPs.^14^ To evaluate their impact on the observed associations between the GSs and our outcomes, we conducted additional analyses by removing *TCF7L2* and *MTNR1B* from the construction of T_GS and G_GS, respectively.

## Results

The descriptive characteristics of the eight cohorts (EFSOCH, Gen3G, HAPO-European, HAPO-Afro-Caribbean, HAPO-East-Asian, HAPO-South-Asian, HAPO-Mexican-American, and FinnGeDi) before and after pregnancy are presented in online supplemental table S2.

### GSs had consistent effects on fasting glucose levels during and after pregnancy

**Figure 1:**
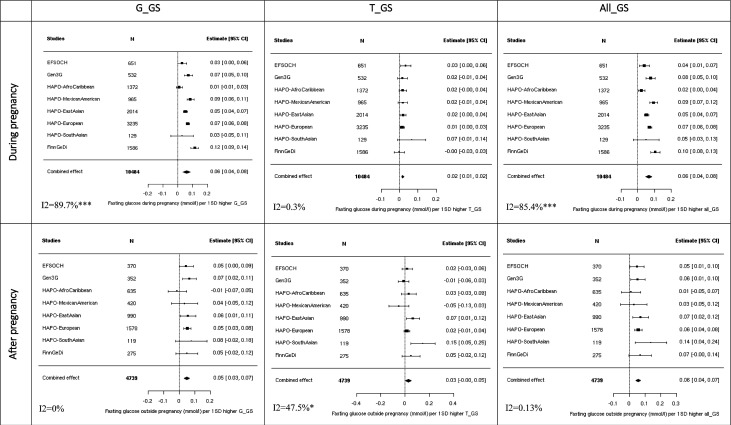
Meta-analysis of the association between fasting glucose and genetic scores. Analyses adjusted solely for principal components and cohort-specific variables (to address unique characteristics of each dataset). Heterogeneity statistics (I^2^) are included in the bottom left of each plot. G_GS=8 SNPs; T_GS=3 SNPs; All_GS=13 SNPs. ***p<0.0001; **p<0.001; *p<0.05. EFSOCH, Exeter Family Study of Childhood Health; FinnGeDi, Finnish Gestational Diabetes; G_GS, variants with stronger effects on GDM than type 2 diabetes; Gen3G, Genetics of Glucose Regulation in Gestation and Growth; GS, genetic score; HAPO, Hyperglycaemia and Adverse Pregnancy Outcome; T_GS, variants with stronger effects on type 2 diabetes than GDM.

In our meta-analyses, both G_GS (8 SNPs) and T_GS (3 SNPs) showed positive associations with fasting glucose during and post pregnancy (β (95% CI) in mmol/L per 1 SD higher GS=0.06 (0.04 to 0.08) during and 0.05 (0.03 to 0.07) post pregnancy for G_GS; 0.02 (0.01 to 0.03) during and 0.02 (–0.0006 to 0.05) post pregnancy for T_GS, figure 1). All_GS (14 SNPs) was also associated with fasting glucose levels both during and post pregnancy with similar effects (0.06 (0.04 to 0.08) vs 0.06 (0.04 to 0.07)). After adjustment for maternal age (online supplemental figure S1), GSs were similarly associated with fasting glucose during pregnancy (0.06 (0.04 to 0.09), 0.02 (0.01 to 0.03) and 0.06 (0.04 to 0.09), respectively, for G_GS, T_GS and All_GS) and after pregnancy (0.06 (0.03 to 0.07), 0.02 (0.0004 to 0.05) and 0.06 (0.04 to 0.07) respectively for G_GS, T_GS and All_GS). Heterogeneity between studies was high for the association estimates during pregnancy (maximum I^2^=86.6% (p<0.0001).

### Associations with 2-hour glucose levels varied according to GSs, with weaker evidence observed after pregnancy

**Figure 2:**
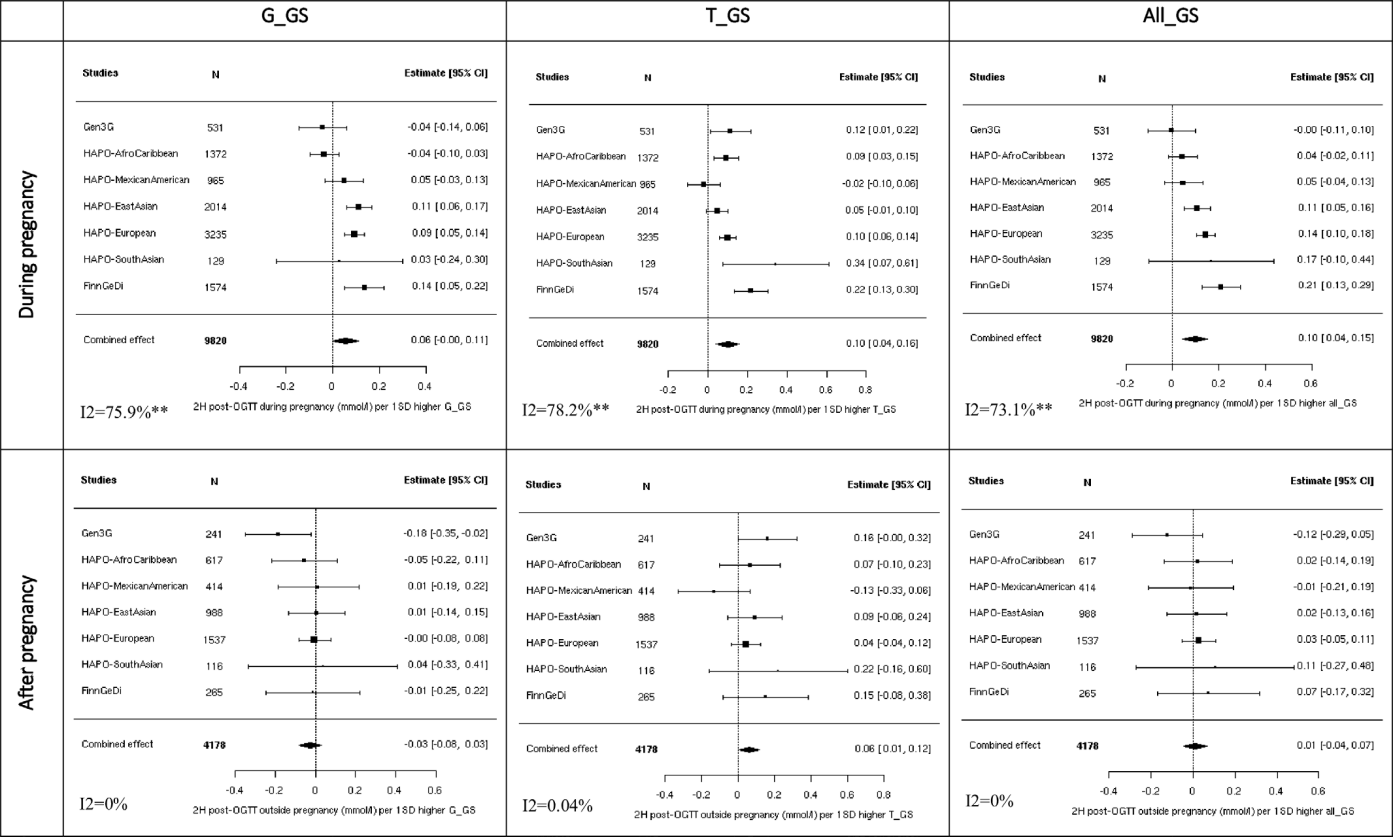
Meta-analysis of the association between 2-hour glucose and GSs. Analyses adjusted solely for principal components and cohort-specific variables (to address unique characteristics of each dataset). Heterogeneity statistics (I^2^) are included in the bottom left of each plot. G_GS=8 SNPs; T_GS=3 SNPs; All_GS=13 SNPs. ***p<0.0001; **p<0.001; *p<0.05. EFSOCH, Exeter Family Study of Childhood Health; FinnGeDi, Finnish Gestational Diabetes; G_GS, variants with stronger effects on GDM than type 2 diabetes; Gen3G, Genetics of Glucose Regulation in Gestation and Growth; GS, genetic score; HAPO, Hyperglycaemia and Adverse Pregnancy Outcome; T_GS, variants with stronger effects on type 2 diabetes than GDM.

G_GS showed weak evidence of association with 2-hour glucose levels during pregnancy (0.06 (−0.002 to 0.11)) and no association with 2-hour glucose levels post pregnancy (−0.03 (−0.08 to 0.03), p>0.05, figure 2). T_GS was associated with 2-hour glucose levels both during and after pregnancy (0.10 (0.04 to 0.16) and 0.06 (0.01 to 0.12), respectively). All_GS was associated with 2-hour glucose levels during pregnancy (0.10 (0.04 to 0.15)) but not afterward (0.01 (−0.04 to 0.07)). After adjusting for maternal age, the results remained consistent: 0.06 (−0.002 to 0.11) during pregnancy and −0.03 (−0.08 to 0.03) post pregnancy for G_GS; 0.11 (0.04 to 0.17) during pregnancy and 0.06 (0.01 to 0.12) post pregnancy for T_GS; and 0.10 (0.04 to 0.16) during pregnancy and 0.01 (−0.05 to 0.07) post pregnancy for All_GS (online supplemental figure S2). Heterogeneity between studies was high for the association estimates during pregnancy (maximum I^2^=78.2%; p=0.001).

### Sensitivity analyses showed consistent results with the main analyses.

The results of the analyses including only women who had had measurements before and after pregnancy were consistent with the results of the main analysis, with wider CIs due to the smaller sample size (online supplemental figures S3–S6).

In the meta-analysis testing the interaction between GSs and pregnancy status, no significant interactions were observed for fasting glucose (online supplemental figure S7). In contrast, we observed significant interactions for 2-hour glucose with the G_GS (−0.097 (-0.154 to −0.041)) and the All_GS (−0.099 (−0.155 to −0.042), indicating that the associations were stronger during pregnancy compared with post pregnancy.

Leave-one-out sensitivity analyses showed that omitting HAPO–Mexican-American strengthened the G_GS association between the G_GS and 2-hour glucose during pregnancy and was the only instance that eliminated heterogeneity.

After removing the *MTNR1B* SNP rs10830963 from the G_GS calculation, associations between G_GS (now containing seven SNPs) and fasting glucose in the meta-analysis remained (0.04 (0.02 to 0.07) during pregnancy and 0.03 (0.01 to 0.04) post pregnancy (online supplemental figure S8). The associations with 2-hour glucose also showed similar patterns to the main analysis, both during and after pregnancy, 0.002 (−0.04 to 0.04) during pregnancy and −0.05 (−0.10 to 0.01) post pregnancy, p>0.05). After removing *TCF7L2* rs34872471 from the T_GS, there was still evidence of association with 2-hour glucose during pregnancy (online supplemental figure S9). However, removal of one SNP from a T_GS (now containing two SNPs) had a large impact on the score’s SD, so it was not possible to make meaningful comparisons between effect sizes in this and the main analysis.

## Discussion

In this meta-analysis of up to 10 225 women from eight international, multiancestral cohorts, we have shown that the combined effects of GDM susceptibility variants on fasting glucose levels were similar during versus after pregnancy. Each of the three GSs tested (G_GS: eight variants with stronger effects on GDM than type 2 diabetes; T_GS: three variants with stronger effects on type 2 diabetes than GDM and All_GS: 14 variants combining G_ and T_GS with two additional unclassified variants) was associated similarly with fasting glucose during and post pregnancy. These findings do not align with our initial hypothesis that G_GS variants would exhibit stronger effects on glucose levels during pregnancy. However, we observed associations with 2-hour glucose during pregnancy, while postpregnancy associations were less pronounced. The All_GS was associated with 2-hour glucose levels during pregnancy but not post pregnancy, and the 95% CIs around the postpregnancy estimate did not include the during-pregnancy estimate (and vice versa), which suggests an overall difference in the combined effect estimate. This pattern appeared to reflect the lack of postpregnancy associations for the G_GS, as the T_GS showed consistent associations with 2-hour glucose levels in both periods.

In the most recent GWAS of GDM from Elliott *et al*, it was suggested that certain SNPs (primarily those in Class-G) may influence glucose homeostasis predominantly during pregnancy.^15^ Our findings did not demonstrate differential effects of the GSs on fasting glucose levels during versus after pregnancy. Variants at the majority of the GDM-associated loci have previously been associated with fasting glucose at genome-wide significance outside of pregnancy, including five out of the eight Class-G loci, two out of the three Class-T loci, and two out of the three unclassified loci.^12^ The Class-G variants include *MTNR1B* rs10830962 and *G6PC2* rs1402837, which are both strongly and consistently associated with fasting glucose outside of pregnancy.^13^ In line with this, a recent analysis of individual SNP effects on fasting glucose in the HAPO study (which contributed to our meta-analysis) found consistent associations for SNPs near *MTNR1B* (rs10830962) and *G6PC2* (rs560887, in LD with rs1402837 (r2=0.1 in Europeans)) during pregnancy and 11–14 years postpartum.^13^ However, a *PCSK1* variant (rs12332295, in LD with the G_GS variant rs1820176; r2=0.4 to 1.0 across multiple ancestries) primarily influenced fasting glucose during pregnancy.^16^ It is possible then that while many of the Class-G variants have consistent effects on fasting glucose during and outside pregnancy, some may have more pronounced effects in gestation. Larger samples are needed for sufficient statistical power to interrogate these individual SNP effects.

We observed differences in the associations with 2-hour glucose levels across the G_GS and All_GS GSs, showing weaker evidence of association after pregnancy. Similarly, the recent analysis of individual SNPs in HAPO examined the differential effects of genetic variants on 2-hour glucose levels post-OGTT during and after pregnancy. Their results showed that the *MTNR1B* variant rs10830962 had a significant impact on 2-hour glucose levels during pregnancy that was not present post pregnancy.^16^ Notably, neither this variant nor the *G6PC2* rs1402837 fasting glucose variant has been robustly associated with 2-hour post-OGTT glucose levels in large GWAS meta-analyses of non-pregnant individuals.^13^ Our results suggest a possible modulation of postprandial glucose regulation during pregnancy, with limited influence on fasting glucose levels. The hormonal and metabolic changes that occur during pregnancy could specifically impact postprandial glucose metabolism by enhancing the effect of certain genetic variants.^16^ However, the influence on fasting glucose may remain more stable, possibly due to different regulatory mechanisms involved in fasting glucose control.

The timing of glucose measurements during pregnancy may represent an important factor to consider in future studies. In the studies contributing to our meta-analysis, glucose levels were measured towards the end of the second and beginning of the third trimester, but results may have been different if measurements were taken earlier, as is sometimes done for high-risk pregnancies in some healthcare settings. Physiologically, pregnancy is associated with a progressive increase in insulin resistance due to hormonal changes, as well as relative β-cell dysfunction, particularly during the second and third trimesters.^17^,^20^ These physiological changes could lead to a decrease in fasting glucose and an increase in postprandial glucose in normoglycemic pregnant women.^17^,^20^ These metabolic adaptations are unique to pregnancy and may alter the expression of genetic effects over time. Some genetic variants may exert consistent effects regardless of gestational stage, while others might be more strongly associated with glucose levels in late pregnancy due to interactions with the evolving hormonal environment.

The differences in diagnostic criteria between GDM and type 2 diabetes may be relevant to the etiology of the GDM variant classifications and our results. GDM diagnosis is characterized by a lower fasting plasma glucose threshold than type 2 diabetes (5.1 mmol/L vs 7.0 mmol/L),^21^ reflecting the importance of maternal fasting glycemia on risk of adverse pregnancy outcomes.^22^ In contrast, although type 2 diabetes is less frequently diagnosed by OGTT than GDM, studies have shown that most individuals diagnosed with type 2 diabetes will have a raised 2-hour glucose ≥11.1 mmol/L.^23 24^ We found that the G_GS was consistently associated with fasting glycemia, whereas the T_GS showed consistent associations with 2-hour glucose levels. This may, in part, relate to the differences in diagnostic criteria for GDM and type 2 diabetes impacting the classification of GDM susceptibility variants.

The strengths of our work are the inclusion of diverse ancestry groups and the large, combined sample size. However, several limitations should be considered. First, we observed substantial heterogeneity between studies, which likely reflects unaccounted differences. Across the HAPO samples, despite the use of standardized protocols, considerable variability in association estimates remained. Part of this heterogeneity may be explained by differences in genetic background. For instance, variation in linkage disequilibrium patterns across ancestries can affect the correlation between index SNPs and causal variants, which may partly explain why estimates in the HAPO-AfroCaribbean cohort tended to be closer to the null compared with those from European ancestry cohorts, where most GDM-associated SNPs were initially identified. The ancestry differences, however, do not account for all heterogeneity. For example, in the analysis of the association between the G_GS and fasting glucose during pregnancy, there was considerable variation among the effect estimates in the EFSOCH, HAPO-European, Gen3G, and FinnGeDi samples, all of which are of northern European ancestry. It is therefore possible that other factors, eg environmental exposures, explain some of the heterogeneity. When we performed the leave-one-out analyses, the heterogeneity did not seem to be consistently driven by any one study (online supplemental table S3). Overall, the high heterogeneity underscores the need for cautious interpretation. A second limitation of our study is that the GSs were derived from Finnish GWAS summary statistics, which may limit applicability to other European or non-European populations. Nonetheless, the included SNPs are well-established loci for glycemic traits, and we verified consistency of allele frequencies across cohorts, reducing the likelihood that LD differences alone explain our findings. Third, the limited availability of postpregnancy follow-up data, particularly for 2-hour glucose measurements across cohorts, reduced the statistical power of our analyses, which may affect the assessment of the persistence of associations, especially between G_GS and all_GS with 2-hour glucose beyond pregnancy. Analyses in larger samples will be important to confirm and extend these findings. A final limitation is the relatively small number of SNPs in the scores (three in Class-T and eight in Class-G), which may make them vulnerable to disproportionate influence by single variants. Indeed, MTNR1B (included in G_GS) and TCF7L2 (included in T_GS) have relatively large effects. However, sensitivity analyses excluding these SNPs showed that they were not solely responsible for the observed associations.

To conclude, GSs associated with GDM have consistent effects on fasting glucose levels during and after pregnancy. This finding reflects the importance of the fasting glucose threshold in GDM diagnosis and indicates biological pathways underlying GDM genetic susceptibility that are not pregnancy specific. However, the associations between predominantly GDM-associated variants and 2-hour glucose levels suggest genetic associations with postprandial glucose may differ in pregnancy. The observed heterogeneity highlights the need to explore factors that may vary between studies or populations.

## Supplementary material

10.1136/bmjdrc-2025-005382 online supplemental file 1

## Data Availability

Data are available upon reasonable request.
