# Supplementary material for: Examining the impact of gestational diabetes genetic susceptibility variants on maternal glucose levels during and post pregnancy
Source: BMJ Open Diabetes Res Care. 2025 Dec 3;13(6):e005382. doi: 10.1136/bmjdrc-2025-005382 (PMC12682201; doi:10.1136/bmjdrc-2025-005382)
Supplement: online supplemental file 1 [file bmjdrc-13-6-s001.docx]

**Supplementary Information**

**Contents:**

1. Information about phenotype and genotype preparation in contributing studies
2. Supplementary Tables (S1, S2) and Figures (S1-S8)
3. References

**Information about phenotype and genotype preparation in contributing studies**

**EFSOCH:** The Exeter Family Study of Childhood Health recruited a total of 986 healthy babies after exclusions, between 1999-2004. A full description of this study was published previously [1]. In this study, fasting blood samples were collected from the mothers during the 28th week of gestation in all women, after a 10-hour overnight fast. The samples were taken in the morning at the parents' homes. Plasma glucose concentrations were subsequently measured at the pathology laboratories of the Royal Devon and Exeter Hospital, using standard reagents provided by the manufacturer and analysed on Modular systems [1, 2]. The same protocol was followed at a median of 5.14 years post-pregnancy, when all women were invited for a post-pregnancy study visit, and a total of 523 women took part [3]. The 2-hour glucose levels post-OGTT were not measured in the EFSOCH study, either during pregnancy or after pregnancy, so EFSOCH only contributed to fasting glucose analyses in the current study.

Genotyping for the entire sample was performed using the Illumina HumanCoreExome-24 array. Raw genotyping data underwent quality control to ensure reliability, with exclusion criteria including a genotype call rate below 95%, SNPs deviating significantly from Hardy-Weinberg equilibrium (p < 1 × 10⁻⁶). Additionally, participants’ phenotypic sex and familial relationships were validated using genotype data analysed with the KING software [4]. Imputation was conducted using the TopMed reference panel via the Michigan Imputation Server. Only SNPs with an imputation quality score above 0.4 and a minor allele frequency exceeding 0.1% were retained for analysis.

For this analysis, the EFSOCH sample included 692 pregnant women and 390 post pregnancy women with genotype and phenotype data available.

**Gen3G:** The Genetics of Glucose Regulation in Gestation and Growth (Gen3G) study is a population-based cohort from Quebec, Canada, which recruited 1,024 pregnant women without diabetes between January 2010 and June 2013. Its primary aim is to enhance understanding of the biological, environmental, and genetic factors influencing glucose regulation during pregnancy and their impact on fetal and offspring development. The details of the cohort description have been published elsewhere [5]. In the Gen3G cohort, glycemia was measured between 24 and 30 weeks’ gestation at fasting and 2-hour post 75 g‑OGTT using the hexokinase method (Roche Diagnostics, Indianapolis, IN) [6, 7]. At 3 and 5 years after delivery, Gen3G participants were invited for a follow-up visiting including collection of fasting blood and a subgroup of women also completed a 75g-OGTT at the 5-year visit with blood samples at fasting and 2h; plasma glucose was measured with the same laboratory methods as for pregnancy samples [8].

Maternal DNA was extracted from blood buffy coats using the Gentra Puregene Blood Kit (Qiagen, Mississauga, Canada) [6]. Genomic data were generated with the Expanded Multi-Ethnic Global Array (Illumina). SNPs included in the analysis had a call rate above 95% and adhered to Hardy-Weinberg equilibrium (p>1×10^-6^). To ensure data accuracy, SNPs were cross-checked for inconsistencies between mother-child pairs or multiple pregnancies from the same individual, and biologically implausible samples were excluded. Imputations were carried out using eagle for phasing, the TOPMed R2 reference panel on TOPMed imputation server using default parameter. Only SNPs with a R2>0.8 were used in the analyses.

For this analysis, only women with complete genomic data and at least one glucose level was included (for the current analyses, 35 women receiving treatment for GDM were excluded), corresponding to 532 women during pregnancy and 352 women outside pregnancy for a total of 533 participants.

**HAPO:** The Hyperglycemia and Adverse Pregnancy Outcome (HAPO) Study is a prospective observational study involving the recruitment of around 25,000 pregnant women across nine countries. The study's details have been documented in prior publications [9, 10]. In the HAPO study, women underwent 75g OGTT between 24 and 32 weeks of pregnancy. Blood samples were collected for fasting glucose analysis, as well as for postprandial glucose measurements at 2 hours post-OGTT. At approximately 10-14 years post pregnancy, participants in the HAPO study were selected according to inclusion criteria: term birth (gestational age ≥ 37 weeks), absence of major neonatal malformations or foetal/neonatal death, caregivers and participants blinded to the initial HAPO study results. Fasting and 2-hour blood glucose (75g OGTT) were measured by the hexokinase method on a Beckman-Coulter SYNCHRON LX analyser in plasma.

The genotyping for the HAPO cohort involved comprehensive data collection across multiple ethnic groups, including European, African Caribbean, East Asian, South Asian, and Mexican American populations. Genomic DNA was extracted from maternal blood samples, and genotyping was performed using platforms such as the Illumina HumanOmni1-Quad BeadChip, or similar, depending on the specific cohort. SNPs had call rates greater than 95%, and their alignment with Hardy-Weinberg equilibrium was confirmed. Imputation was carried out against the TOPMed reference panel on the TOPMed imputation server. The detailed methodology of genotyping in the HAPO study, including how these specific ethnic analyses were carried out, has been published elsewhere [11–16].

In our study, analyses were performed separately for each HAPO subgroup. These subgroups consisted of 1372, 965, 2014, 3235, and 129 women during pregnancy; and 636, 421, 990, 1580, and 119 women post pregnancy, representing African Caribbean, Mexican American, East Asian, European, and South Asian ancestries, respectively.

**FinnGeDi :** The Finnish Gestational Diabetes Study (FinnGeDi) is a case-control study conducted within the Finnish population to investigate genetic and environmental factors associated with the development of gestational diabetes. Previously described in detail, this study recruited a total of 1,066 non-diabetic pregnant women and 1,146 women diagnosed with gestational diabetes, between 2009 and 2012 [17, 18]. Blood samples were taken to measure fasting glucose levels, as well as glucose levels at 2 hours following 75g OGTT, typically conducted between 24 and 28 weeks of gestation according to the Finnish National current Care Guidelines. Glucose data were collected both during pregnancy and consistently 10-15 years post pregnancy. Postpartum samples were collected from women who participated in the follow-up visit 11-15 years after the index pregnancy. All women who had taken part in the original study and were residing in the Oulu region were invited, without any additional selection criteria. However, women who attended the follow-up visit were slightly older, and had somewhat higher prepregnancy BMI and socioeconomic status during their index pregnancy compared with non-participants. Women with type 1 (n=1) or type 2 (n=15) diagnosed before the follow-up visit were excluded from the postpartum analyses, as they were using glucose-lowering medications and did not have 2-hour glucose data available (the OGTT was not performed in women with pre-existing diabetes).

The FinnGeDi genetic data were divided into two datasets because they were genotyped at different times. Maternal DNA was extracted from venous blood samples. Genotyping was conducted using Illumina Infinium Omni 2.5-8 for a subset of 516 samples and Illumina GSA 500k for a subset of 1455 samples. After quality control, variants with SNP-based call rate > 95% or Hardy-Weinberg equilibrium p > 0.0001 were included. Datasets were imputed separately using Finnish ancestry reference-panel SISu v3 (protocol: <https://www.protocols.io/view/genotype-imputation-workflow-v3-0-e6nvw78dlmkj/v2>). Eagle v2.3.5 was used for the genotype phasing and Beagle v4.1 was used for the imputation.

GS calculations were performed separately for each genotyping datasets and then combined in the regression analyses. A total of 1586 women with fasting, and 2-hour glucose data, along with genotype information during pregnancy, were included in our analysis. After pregnancy, 275 women had fasting and 2-hour glucose measurements.

**Supplementary Tables and Figures**

| Chromosome | Position, hg38 | rsid | Effect allele | other allele | Nearest gene | Class | Effect size (SE) | EFSOCH | Gen3G | HAPO-AFR | HAPO-AMR | HAPO-EAS | HAPO-EUR | HAPO-SAS | FinnGeDi | FinnGeDi |
| --- | --- | --- | --- | --- | --- | --- | --- | --- | --- | --- | --- | --- | --- | --- | --- | --- |
| 2 | 27519736 | Rs780093 | C | T | GCKR | G | 0.12 (0.01) | ✓ | ✓ | ✓ | ✓ | ✓ | ✓ | ✓ | ✓ | ✓ |
| 2 | 168900844 | Rs1402837 | T | C | SPC25-G6PC2 | G | 0.11 (0.02) | ✓ | ✓ | ✓ | ✓ | ✓ | ✓ | ✓ | ✓ | ✓ |
| 3 | 123376465 | Rs6798189 | G | A | ADCY5 | Unclassified | 0.1 (0.02) | ✓ | ✓ | ✓ | ✓ | ✓ | ✓ | ✓ | ✓ | ✓ |
| 5 | 96360881 | Rs1820176 | T | C | PCSK1 | G | 0.14 (0.01) | ✓ | ✓ | ✓ | ✓ | ✓ | ✓ | ✓ | ✓ | ✓ |
| 6 | 20676183 | Rs34499031 | TAA | T | CDKAL1 | T | 0.12 (0.01) | ✓ | ✓ | ✓ | ✓ | ✓ | ✓ | ✓ | ✓ | ✓ |
| 6 | 151805650 | Rs537224022 | C | G | ESR1 | G | 0.45 (0.08) | ✓ | NA | NA | ✓ | NA | ✓ | ✓ | ✓ | ✓ |
| 9 | 22136490 | Rs1333051 | A | T | CDKN2B | Unclassified | 0.13 (0.02) | ✓ | ✓ | ✓ | ✓ | ✓ | ✓ | ✓ | ✓ | ✓ |
| 9 | 22134303 | Rs7019437 | G | C | CDKN2B | Unclassified | 0.04 (0.01) | ✓ | ✓ | NA | NA | NA | NA | NA | ✓ | ✓ |
| 10 | 112994312 | Rs34872471 | C | T | TCF7L2 | T | 0.17 (0.02) | ✓ | ✓ | ✓ | ✓ | ✓ | ✓ | ✓ | ✓ | ✓ |
| 11 | 92975544 | Rs10830963 | G | C | MTNR1B | G | 0.4 (0.01) | ✓ | ✓ | ✓ | ✓ | NA | ✓ | ✓ | ✓ | ✓ |
| 12 | 4275678 | Rs76895963 | T | G | CCND2 | T | 0.26 (0.04) | ✓ | ✓ | ✓ | ✓ | ✓ | ✓ | ✓ | ✓ | ✓ |
| 12 | 97457224 | Rs74628648 | C | T | NEDD1 | G | 0.17 (0.03) | ✓ | ✓ | ✓ | ✓ | ✓ | ✓ | ✓ | ✓ | ✓ |
| 16 | 81488676 | Rs2926003 | C | T | CMIP | G | 0.08 (0.02) | ✓ | ✓ | ✓ | ✓ | ✓ | ✓ | ✓ | ✓ | ✓ |
| X | 19380197 | Rs56381411 | C | T | MAP3K15 | G | 0.4 (0.06) | ✓ | NA | NA | NA | NA | NA | NA | ✓ | ✓ |

**Supplementary Table S1: Availability of SNPs across cohorts**

✓ = SNP available in the cohort NA = SNP missing in the cohort

**Supplementary Table S2: Descriptions (mean (sd)) of the characteristics of each dataset**

|  | **EFSOCH** | **Gen3G** | **HAPO-AFR** | **HAPO-AMR** | **HAPO-EAS** | **HAPO-EUR** | **HAPO-SAS** | **FinnGeDi** |
| --- | --- | --- | --- | --- | --- | --- | --- | --- |
|  | **N=692** | **N=532** | **N=1372** | **N=965** | **N=2014** | **N=3235** | **N=129** | **N=1586** |
| **G_GS** | 2.74 (0.30) | 1.02 (0.30) | 0.93(0.19) | 1.91 (0.28) | 1.22 (0.32) | 1.94 (0.31) | 1.99 (0.33) | 2.83 (0.34) |
| **T_GS** | 0.67(0.13) | 0.68 (0.14) | 0.79 (0.14) | 0.66 (0.13) | 0.09 (0.09) | 0.67 (0.14) | 0.71 (0.14) | 0.66 (0.13) |
| **All_GS** | 3.82 (0.34) | 2.11 (0.33) | 2.13 (0.25) | 2.94 (0.32) | 1.73 (0.34) | 2.98 (0.34) | 3.09 (0.36) | 3.93 (0.37) |
| **Description of variables during pregnancy** | | | | | | | | |
| **Gestational age at glucose measurement (weeks)** | 28* | 26.5 (1.0) | 27.1 (1.7) | 27.0 (2) | 28.0 (1.6) | 28.4 (1.4) | 28.4 (1.2) | 24.6 (6.3) |
| **Maternal age at glucose measurement (years)** | 30.5 (5.2) | 28.9 (4.1) | 25.7 (5.7) | 28.9 (5.4) | 29.2 (5.5) | 31.2 (5.2) | 29.4 (4.7) | 30.9 (5.3) |
| **Fasting glucose (mmol/l)** | 4.3 (0.4) | 4.2 (0.3) | 4.5 (0.4) | 4.6 (0.4) | 4.4 (0.3) | 4.5 (0.4) | 4.7 (0.4) | 5.0 (0.6) |
| **2-h glucose (mmol/l)** | NA | 5.7 (1.2) | 6.0 (1.2) | 6.1 (1.3) | 6.5 (1.3) | 6.0 (1.2) | 6.5 (1.6) | 6.9 (1.7) |
| **Description of variables outside pregnancy** | | | | | | | | |
|  | **N=390** | **N=352** | **N=636** | **N=421** | **N=990** | **N=1580** | **N=119** | **N=275** |
| **Maternal age at glucose measurement (years)** | 36.7 (5.0) | 34.6 (4.1) | 38.0 (5.9) | 41.1 (5.5) | 41.7 (4.9) | 43.8 (4.9) | 41.2 (4.8) | 45.1 (5.2) |
| **Fasting glucose (mmol/l)** | 4.6 (0.4) | 4.7 (0.4) | 5.0 (0.8) | 5.2 (0.8) | 5.2 (0.8) | 5.1 (0.5) | 5.2 (0.6) | 5.5 (0.6) |
| **2-h glucose (mmol/l)** | NA | 5.2 (1.3) | 6.5 (2.2) | 6.6 (2) | 6.7 (2.3) | 6.0 (1.6) | 6.8 (2) | 6.0 (1.9) |

* Fasting glucose during pregnancy in EFSOCH samples were taken at 28 weeks' gestation

**Supplementary Table S3: Leave-One-Out study using the main analyses model, Impact of Cohort Exclusion on Heterogeneity (I²)**

|  |  | Fasting glucose during pregnancy | | Fasting glucose after pregnancy | | 2-hour glucose during pregnancy | | 2-hour glucose after pregnancy | |
| --- | --- | --- | --- | --- | --- | --- | --- | --- | --- |
| GS | ExcludedStudy | I2 | Pvalue_I2 | I2 | Pvalue_I2 | I2 | Pvalue_I2 | I2 | Pvalue_I2 |
| G_GS | EFSOCH | 90.49 | 1.07E-08 | 0 | 0.49 | NA | NA | NA | NA |
|  | HAPO-Afro-Caribbean | 91.50 | 2.47E-09 | 0 | 0.56 | 73.06 | 0.004 | 0 | 0.994 |
|  | HAPO-Mexican-American | 83.40 | 2.27E-04 | 0 | 0.99 | 53.39 | 0.093 | 0 | 0.489 |
|  | HAPO-East-Asian | 90.52 | 1.62E-08 | 0 | 0.50 | 79.89 | 0.001 | 1.17 | 0.499 |
|  | HAPO-European | 89.48 | 2.95E-09 | 0 | 0.50 | 73.46 | 0.002 | 8.41 | 0.508 |
|  | HAPO-South-Asian | 88.82 | 7.24E-09 | 0 | 0.51 | 73.20 | 0.002 | 2.17 | 0.556 |
|  | Gen3G | 91.41 | 2.01E-09 | 0 | 0.53 | 79.03 | 0.001 | 0 | 0.491 |
|  | FinnGeDi | 85.25 | 3.61E-06 | 0 | 0.49 | 74.67 | 0.002 | 0 | 0.477 |
| T_GS | EFSOCH | 3.16 | 0.737 | 63.09 | 0.02 | NA | NA | NA | NA |
|  | HAPO-Afro-Caribbean | 0.34 | 0.568 | 39.35 | 0.06 | 83.83 | 0.001 | 0.01 | 0.402 |
|  | HAPO-Mexican-American | 0.58 | 0.568 | 62.34 | 0.02 | 82.71 | 0.001 | 10.68 | 0.242 |
|  | HAPO-East-Asian | 0.16 | 0.570 | 43.32 | 0.07 | 67.81 | 0.016 | 0 | 0.752 |
|  | HAPO-European | 3.30 | 0.585 | 0.36 | 0.07 | 79.60 | 0.002 | 6.44 | 0.255 |
|  | HAPO-South-Asian | 0.02 | 0.600 | 58.15 | 0.03 | 79.85 | 0.001 | 19.51 | 0.274 |
|  | Gen3G | 0 | 0.786 | 0.02 | 0.20 | 78.33 | 0.003 | 0.03 | 0.300 |
|  | FinnGeDi | 0.01 | 0.813 | 57.08 | 0.03 | 51.06 | 0.033 | 0.03 | 0.294 |
| all_GS | EFSOCH | 86.64 | 2.24E-06 | 0.20 | 0.49 | NA | NA | NA | NA |
|  | HAPO-Afro-Caribbean | 87.73 | 1.15E-06 | 0.18 | 0.48 | 69.93 | 0.013 | 0 | 0.993 |
|  | HAPO-Mexican-American | 75.93 | 0.002 | 1.36 | 0.77 | 72.92 | 0.013 | 0 | 0.685 |
|  | HAPO-East-Asian | 84.83 | 1.81E-05 | 0.11 | 0.52 | 76.16 | 0.005 | 0 | 0.688 |
|  | HAPO-European | 84.79 | 2.46E-06 | 0.50 | 0.52 | 76.55 | 0.002 | 0 | 0.682 |
|  | HAPO-South-Asian | 84.36 | 2.33E-06 | 0.0001 | 0.48 | 70.36 | 0.010 | 0 | 0.731 |
|  | Gen3G | 87.80 | 7.42E-07 | 0 | 0.81 | 77.43 | 0.002 | 0 | 0.719 |
|  | FinnGeDi | 82.49 | 4.97E-05 | 0.677 | 0.49 | 61.03 | 0.025 | 0 | 0.723 |

|  | **G_GS** | **T_GS** | **All_GS** |
| --- | --- | --- | --- |
| **During pregnancy** | 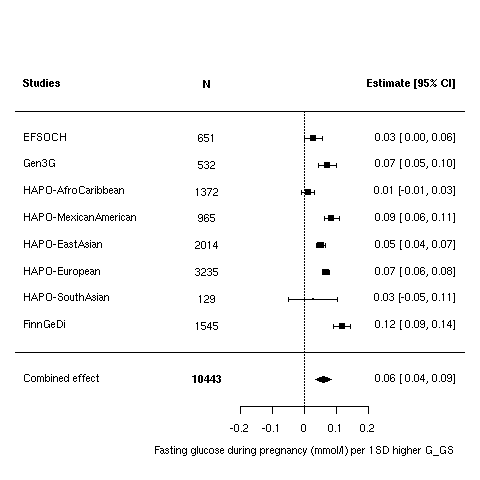  I2=90.5%*** | 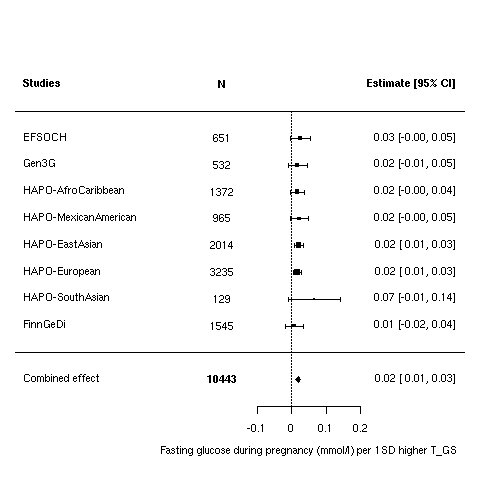  I2=0.11% | 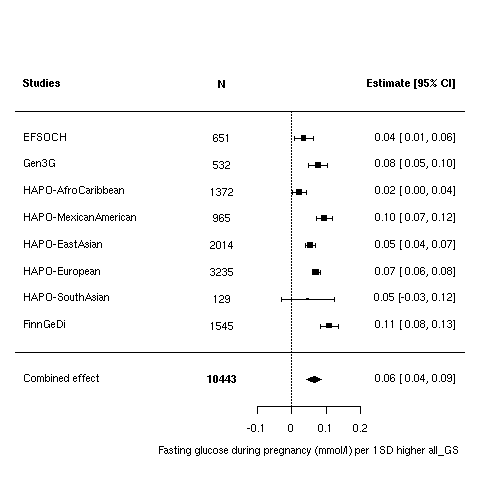  I2=87.8%*** |
| **After pregnancy** | 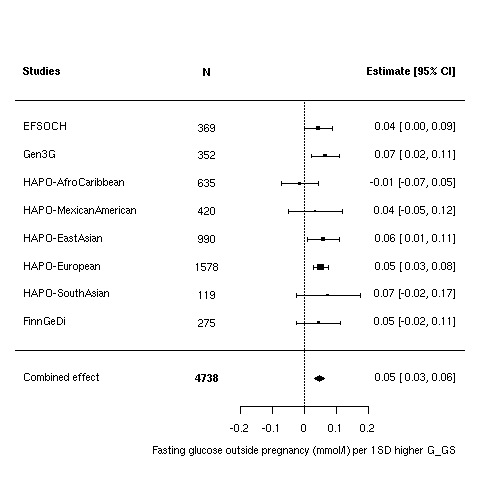  I2=0.57% | 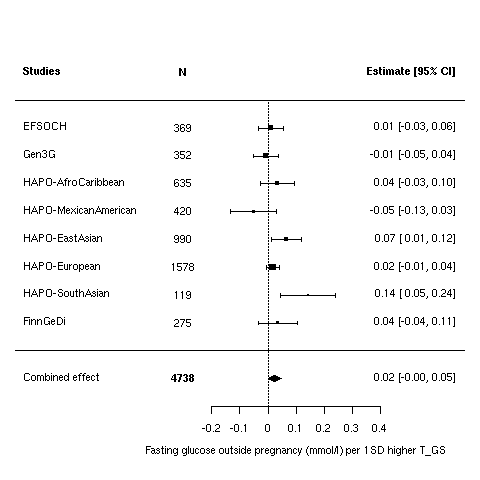  I2=40.3%* | 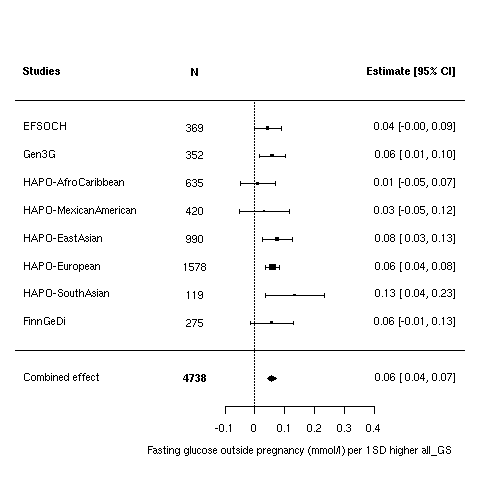  I2=0.05% |

**Supplementary figure S1: Meta-analysis of the association between fasting glucose and genetic scores, adjusted for maternal age at glucose measurement**

Analyses also adjusted for principal components and cohort-specific variables (to address unique characteristics of each dataset). Heterogeneity statistics (I^2^) are included in the bottom left of each plot.***: p-value <.0001; **: p-value < 0.001; *: p-value < 0.05

|  | **G_GS** | **T_GS** | **All_GS** |
| --- | --- | --- | --- |
| **During pregnancy**  I2=76.3%** | 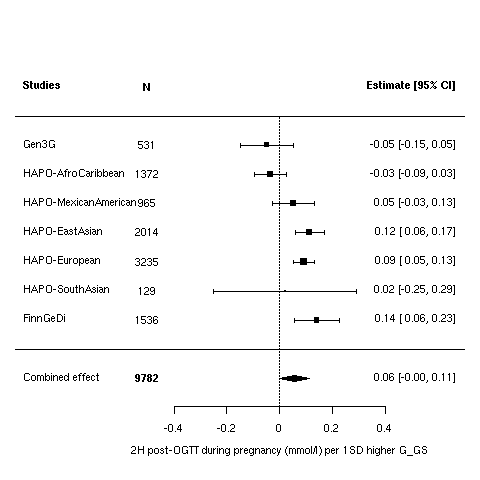 | 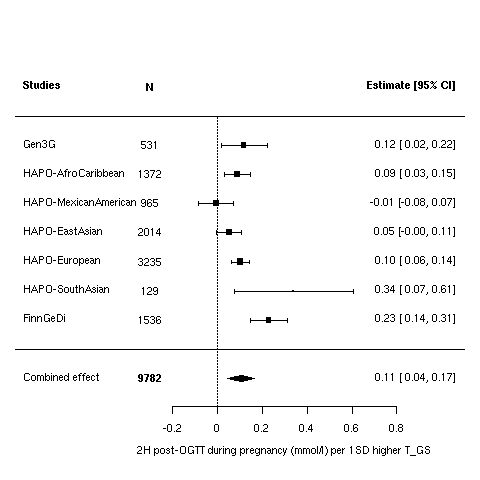  I2=79%** | 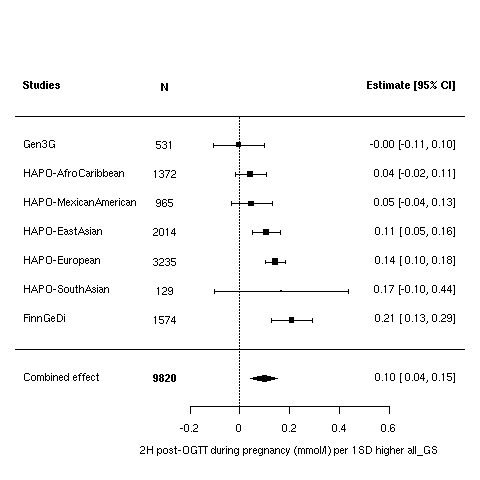  I2=75.7%** |
| **After pregnancy** | 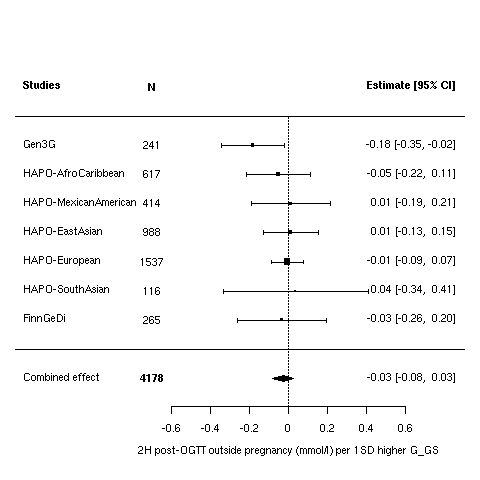  I2=0% | 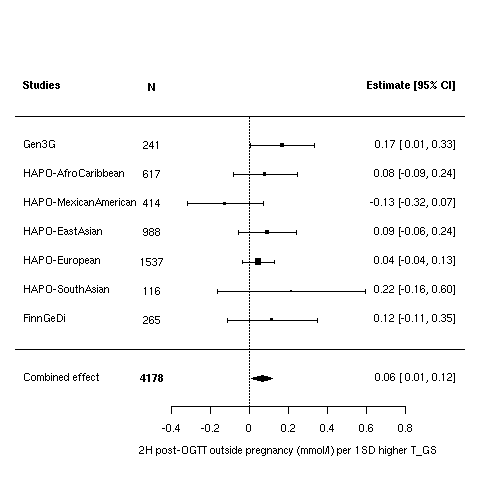  I2=0.09% | 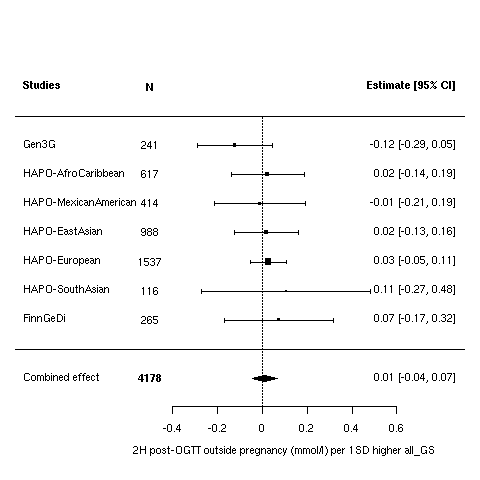  I2=0% |

**Supplementary Figure S2: Meta-analysis of the association between 2-hour glucose and genetic scores, adjusted for maternal age at glucose measurement**

Analyses adjusted also for principal components and cohort-specific variables (to address unique characteristics of each dataset). Heterogeneity statistics (I^2^) are included in the bottom left of each plot.***: p-value <.0001; **: p-value < 0.001; *: p-value < 0.05

|  | **G_GS** | **T_GS** | **All_GS** |
| --- | --- | --- | --- |
| **During pregnancy** | 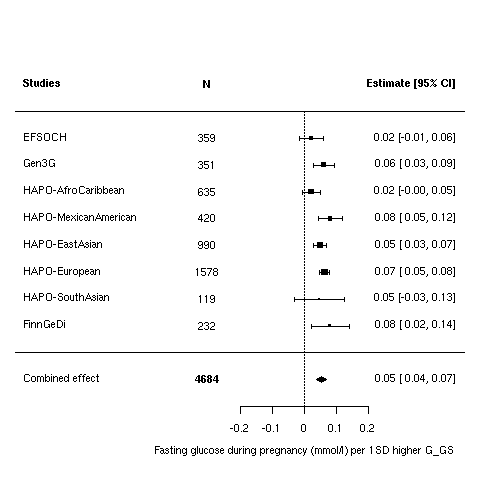  I2=48.6% | 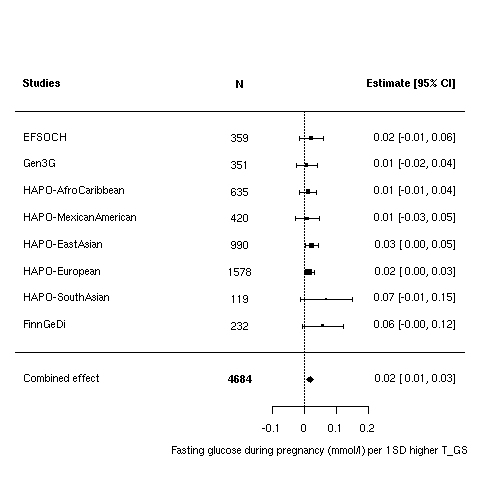  I2=0.32% | 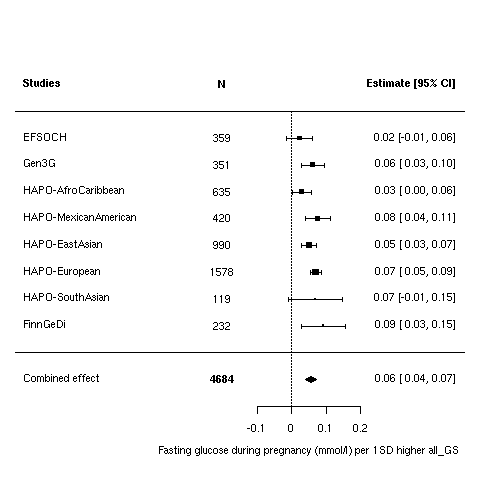  I2=42.1% |
| **After pregnancy** | 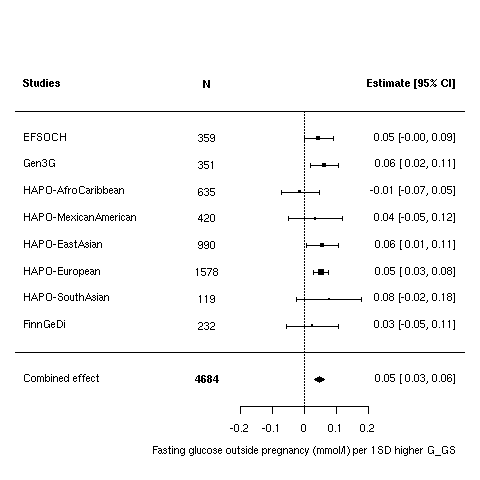  I2=0% | 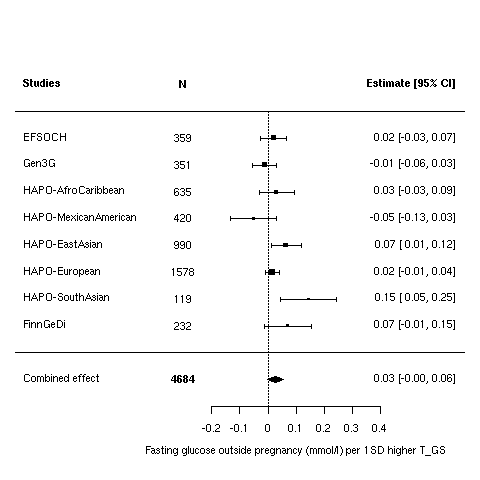  I2=54.1%* | 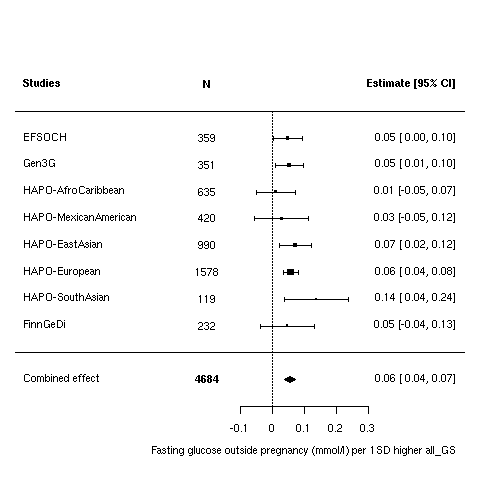  I2=0.01% |

**Supplementary Figure S3: Meta-analysis of the association between fasting glucose and genetic scores, including only women who had measurements before and after pregnancy.** Analyses adjusted solely for principal components and cohort-specific variables (to address unique characteristics of each dataset). Heterogeneity statistics (I^2^) are included in the bottom left of each plot.

***: p-value <.0001; **: p-value < 0.001; *: p-value < 0.05

|  | **G_GS** | **T_GS** | **All_GS** |
| --- | --- | --- | --- |
| **During pregnancy** | 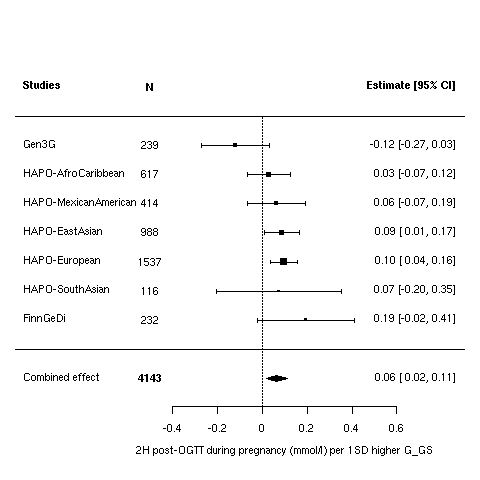  I2=22.3% | 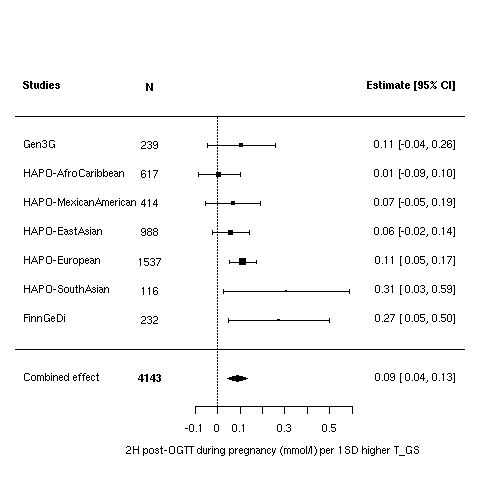  I2=16.7% | 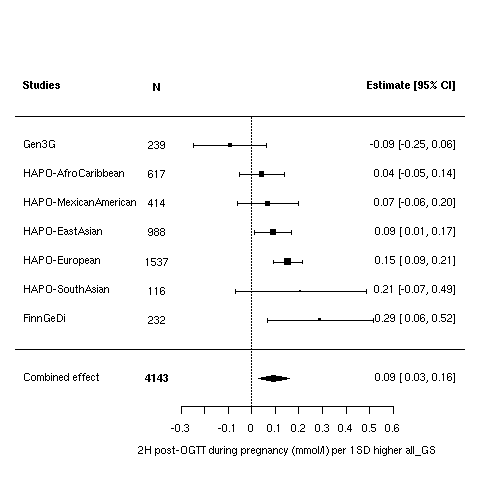  I2=54.5%* |
| **After pregnancy** | 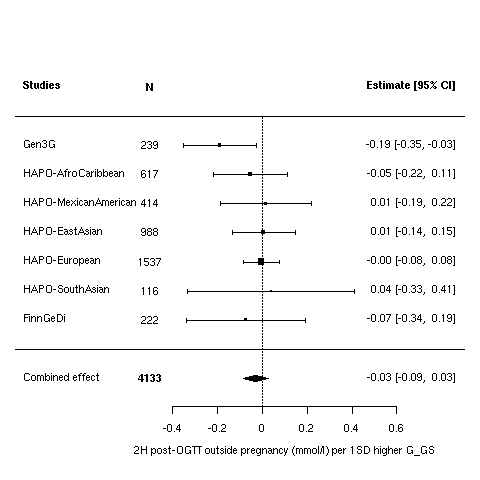  I2=0% | 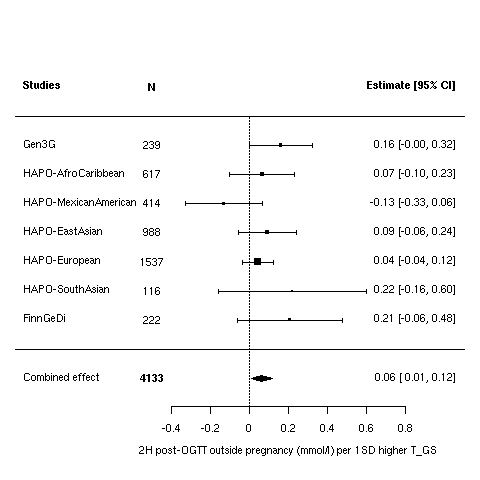  I2=0.05% | 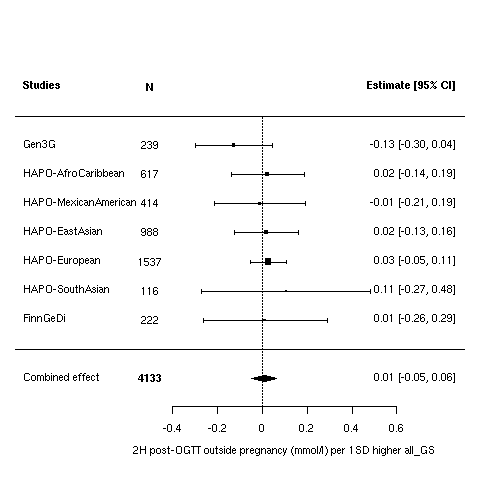  I2=0% |

**Supplementary Figure S4: Meta-analysis of the association between 2-hour glucose and genetic scores, including only women who had measurements before and after pregnancy.** Analyses adjusted solely for principal components and cohort-specific variables (to address unique characteristics of each dataset). Heterogeneity statistics (I^2^) are included in the bottom left of each plot.

***: p-value <.0001; **: p-value < 0.001; *: p-value < 0.05

|  | **G_GS** | **T_GS** | **All_GS** |
| --- | --- | --- | --- |
| **During pregnancy** | 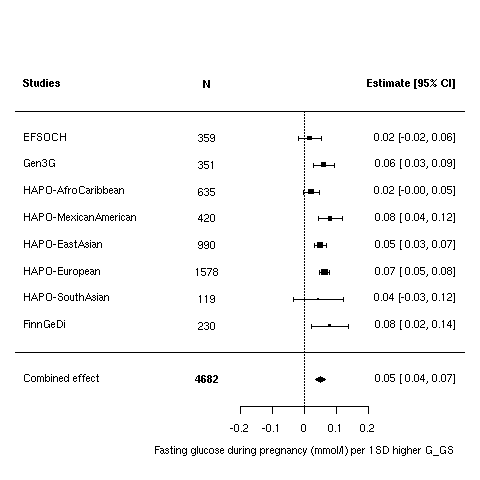  I2=54.7%* | 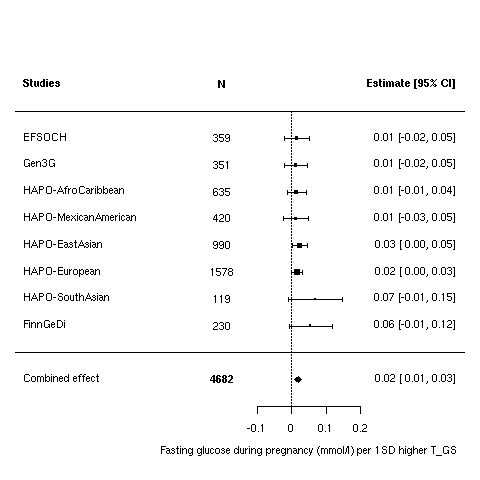  I2=0% | 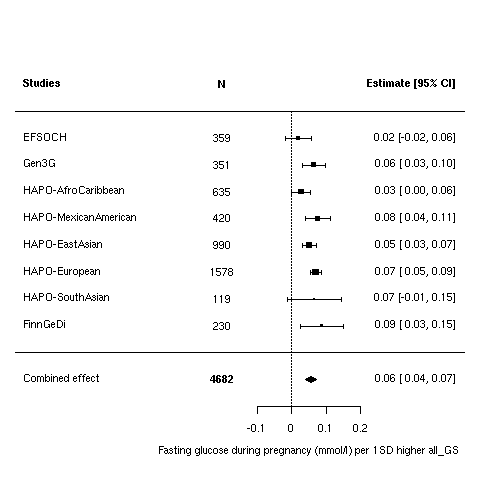  I2=51.6%* |
| **After pregnancy** | 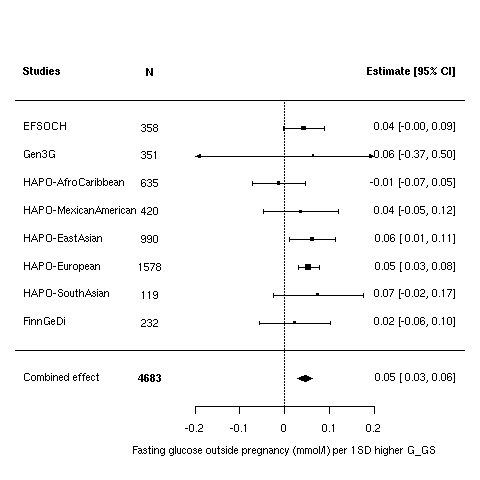  I2=0% | 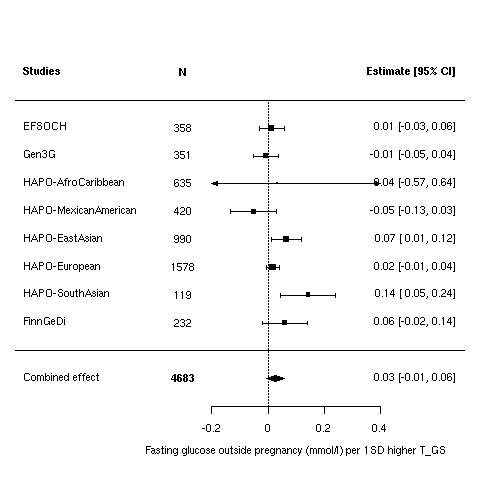  I2=58.8%* | 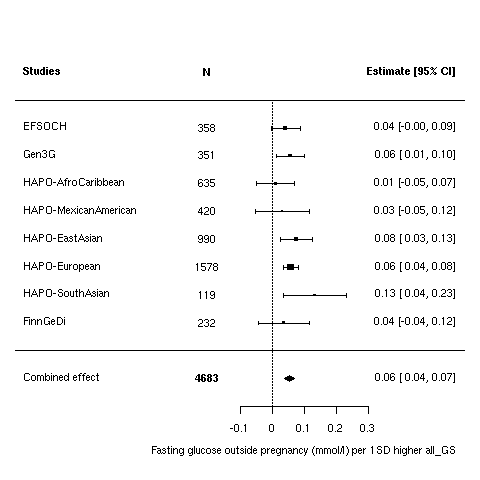  I2=0.74% |

**Supplementary Figure S5: Meta-analysis of the association between fasting glucose and genetic scores, including only women who had measurements before and after pregnancy and adjusted for maternal age at glucose.** Analyses adjusted also for principal components and cohort-specific variables (to address unique characteristics of each dataset). Heterogeneity statistics (I^2^) are included in the bottom left of each plot.

***: p-value <.0001; **: p-value < 0.001; *: p-value < 0.05

|  | **G_GS** | **T_GS** | **All_GS** |
| --- | --- | --- | --- |
| **During pregnancy** | 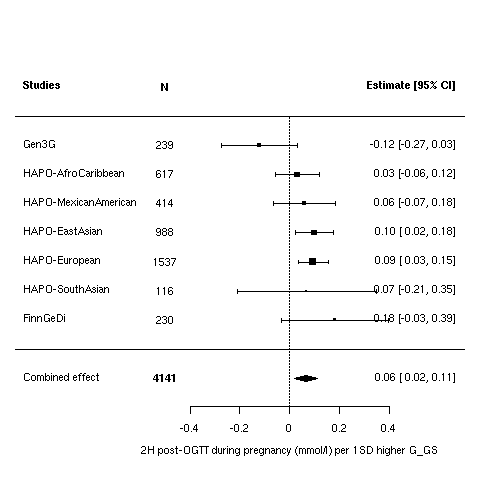  I2=24.7% | 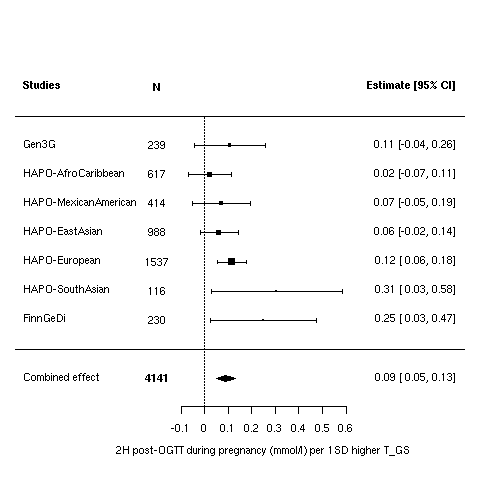  I2=8.2% | 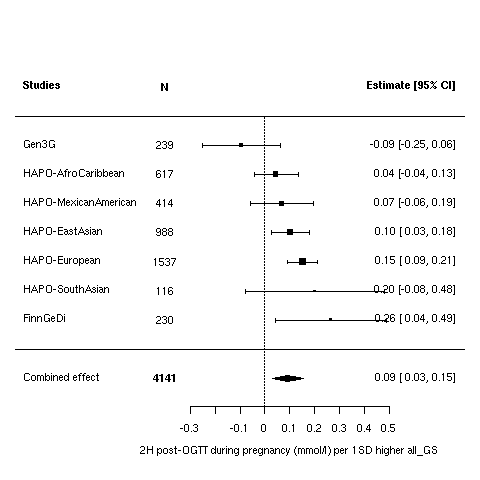  I2=51.7%* |
| **After pregnancy** | 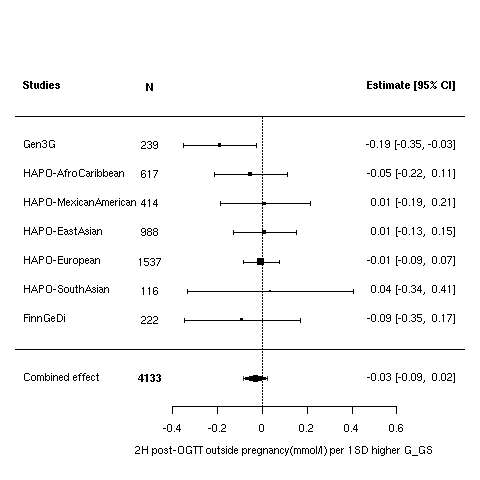  I2=0% | 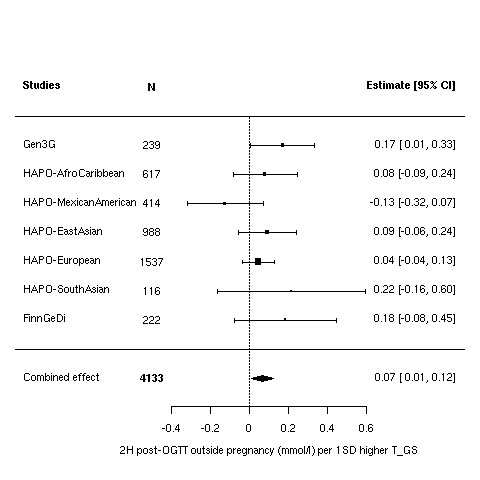  I2=0.03% | 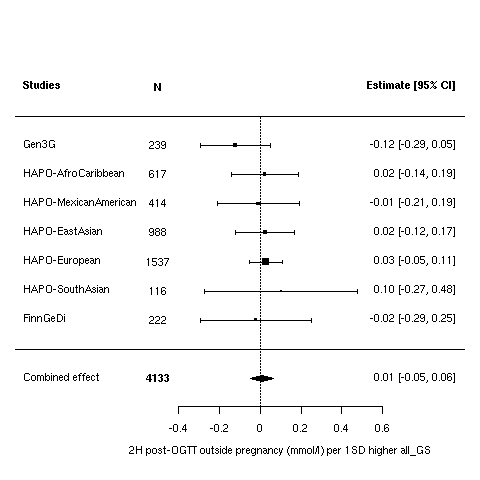  I2=0% |

**Supplementary Figure S6: Meta-analysis of the association between 2-hour glucose and genetic scores, including only women who had measurements before and after pregnancy and adjusted for maternal age at glucose measurement.** Analyses adjusted also for principal components and cohort-specific variables (to address unique characteristics of each dataset). Heterogeneity statistics (I^2^) are included in the bottom left of each plot.

***: p-value <.0001; **: p-value < 0.001; *: p-value < 0.05

|  | **G_GS** | **T_GS** | **All_GS** |
| --- | --- | --- | --- |
| **Fasting glucose** | 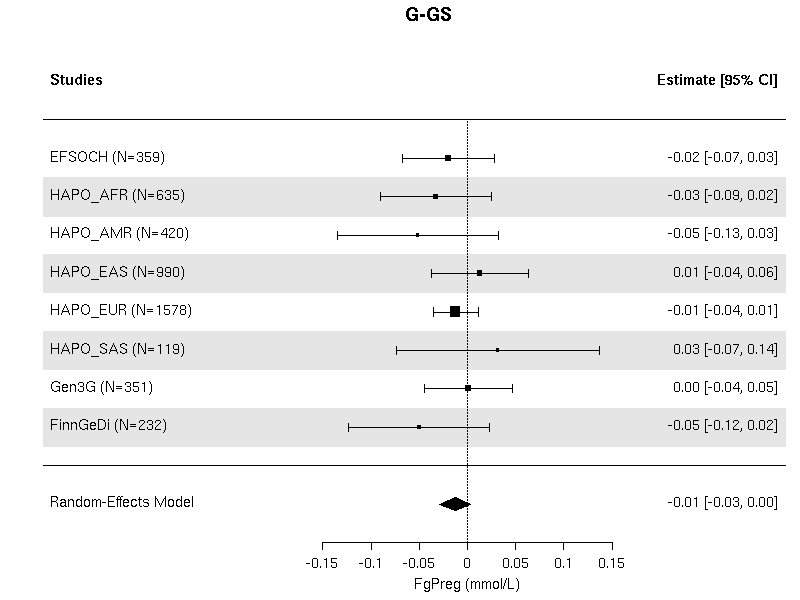 | 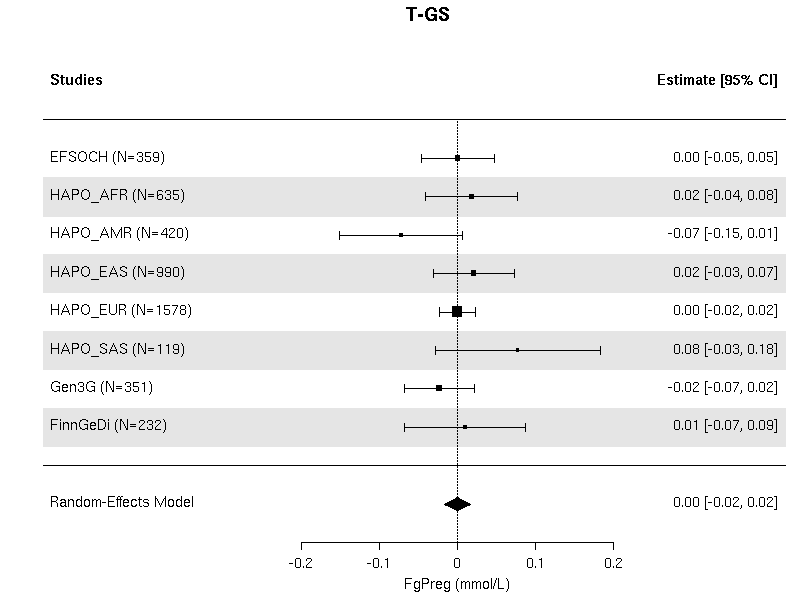 | 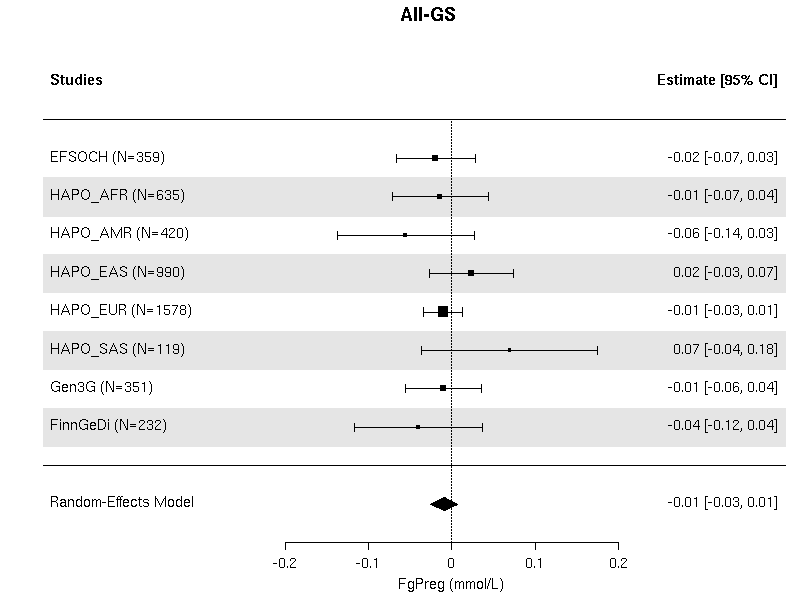 |
| **2-hour post-OGTT** | 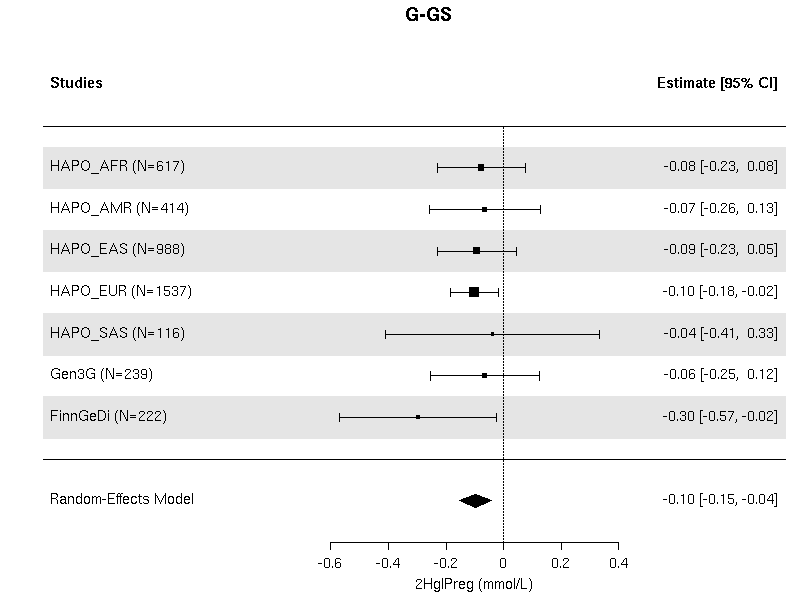 | 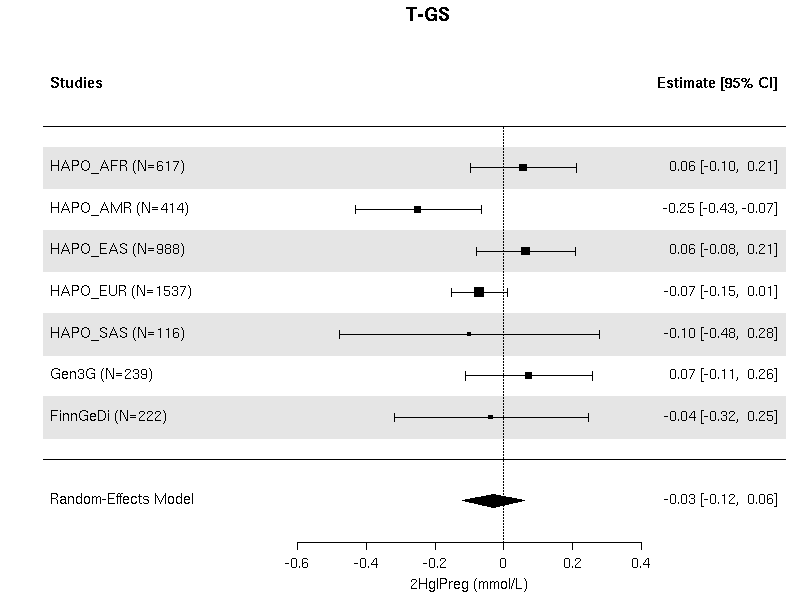 | 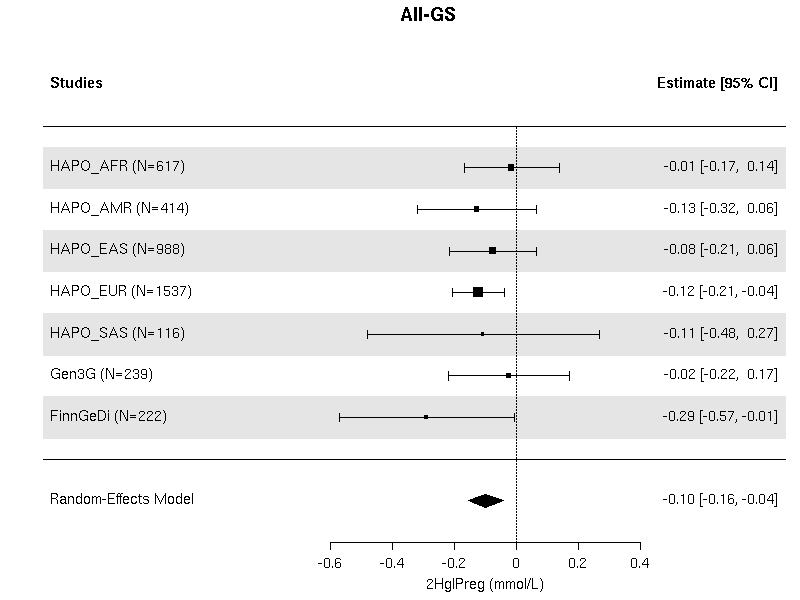 |

**Supplementary figure S7: Forest plot showing cohort-specific and pooled estimates for the interaction between the genetic scores and pregnancy status** (**in GS × PregStatus)** **in relation to fasting glucose and 2-hour glucose levels (mmol/L).**
*Estimates (β) represent the difference in the effect of the genetic score on fasting glucose or 2-hour glucose post-pregnancy compared with during pregnancy reference). **Models were adjusted for ancestry principal components, and cohort-specific covariates (e.g., centre, batch).

|  | **Fasting glucose** | **2-hour post-OGTT** |
| --- | --- | --- |
| **During pregnancy** | 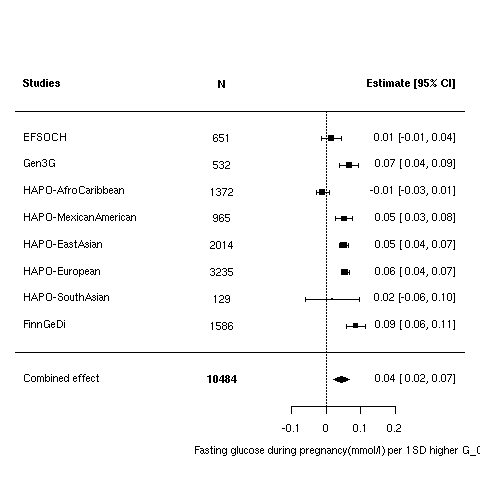  I2=88.2%*** | 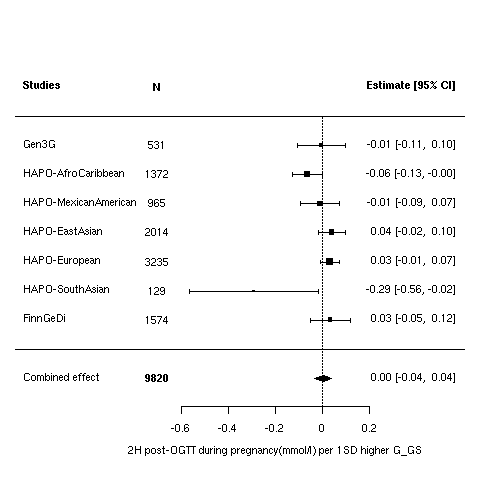  I2=42.8%* |
| **After pregnancy** | 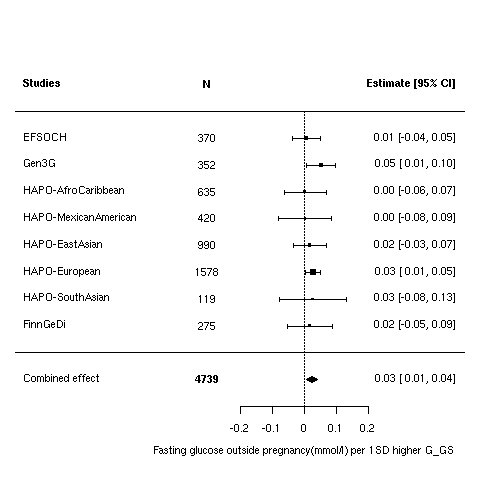  I2=0% | 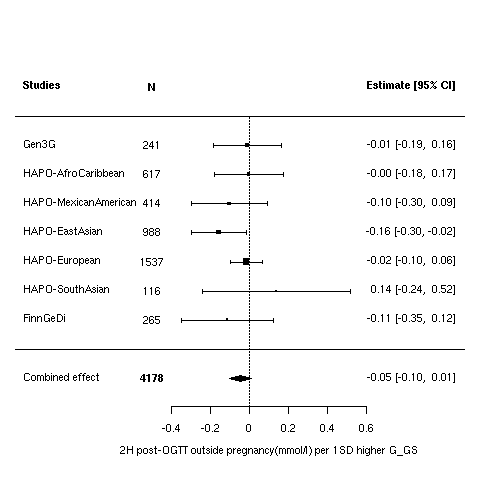  I2=0.99% |

**Supplementary Figure S8: Meta-analysis of the association between glucose levels (fasting glucose and 2-hour glucose post-OGTT) and G_GS, removing *MTNR1B***

Analyses adjusted solely for principal components and cohort-specific variables (to address unique characteristics of each dataset)

***: p-value <.0001; **: p-value < 0.001; *: p-value < 0.05

|  | **Fasting glucose** | **2-hour post-OGTT** |
| --- | --- | --- |
| **During pregnancy** | 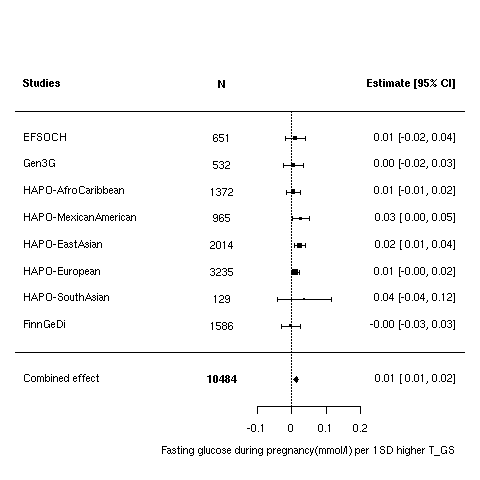  I2=0% | 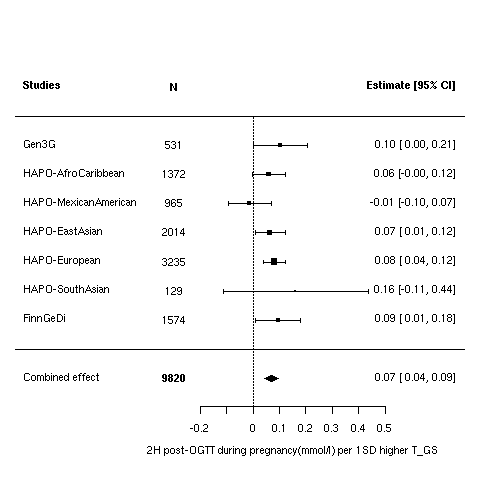  I2=0% |
| **After pregnancy** | 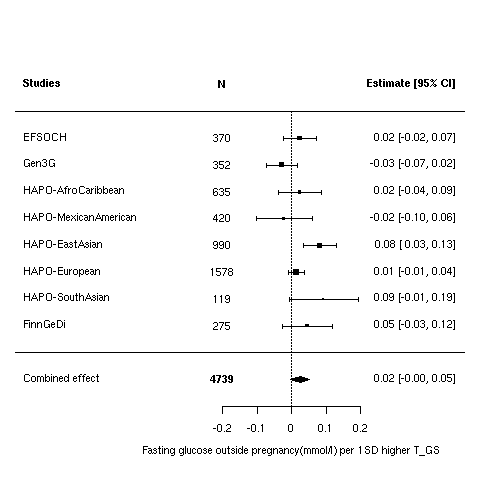  I2=54.8%* | 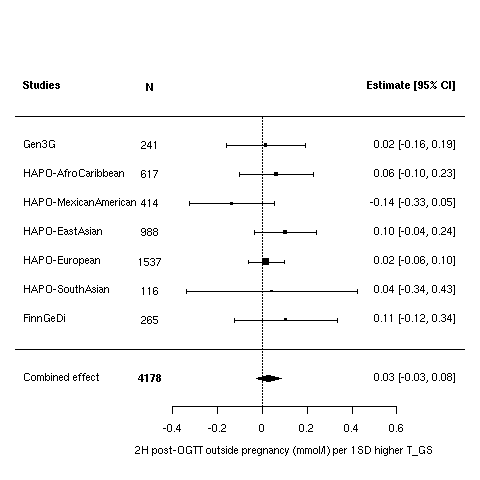  I2=0% |

**Supplementary Figure S9: Meta-analysis of the association between glucose levels (fasting glucose and 2-hour glucose post-OGTT) and T_GS, removing *TCF7L2***

* Analyses adjusted solely for principal components and cohort-specific variables (to address unique characteristics of each dataset)

***: p-value <.0001; **: p-value < 0.001; *: p-value < 0.05

**References**

1. Knight B, Shields BM, Hattersley AT (2006) The Exeter Family Study of Childhood Health (EFSOCH): study protocol and methodology. Paediatric and Perinatal Epidemiology 20(2):172–179. https://doi.org/10.1111/j.1365-3016.2006.00701.x

2. Weedon MN, Frayling TM, Shields B, et al (2005) Genetic Regulation of Birth Weight and Fasting Glucose by a Common Polymorphism in the Islet Cell Promoter of the Glucokinase Gene. Diabetes 54(2):576–581. https://doi.org/10.2337/diabetes.54.2.576

3. Shields BM, Knight BA, Hill AV, Hattersley AT, Vaidya B (2013) Five-Year Follow-Up for Women With Subclinical Hypothyroidism in Pregnancy. J Clin Endocrinol Metab 98(12):E1941–E1945. https://doi.org/10.1210/jc.2013-2768

4. Manichaikul A, Mychaleckyj JC, Rich SS, Daly K, Sale M, Chen W-M (2010) Robust relationship inference in genome-wide association studies. Bioinformatics 26(22):2867–2873. https://doi.org/10.1093/bioinformatics/btq559

5. Guillemette L, Allard C, Lacroix M, et al (2016) Genetics of Glucose regulation in Gestation and Growth (Gen3G): a prospective prebirth cohort of mother–child pairs in Sherbrooke, Canada. BMJ Open 6(2):e010031. https://doi.org/10.1136/bmjopen-2015-010031

6. Powe CE, Nodzenski M, Talbot O, et al (2018) Genetic Determinants of Glycemic Traits and the Risk of Gestational Diabetes Mellitus. Diabetes 67(12):2703–2709. https://doi.org/10.2337/db18-0203

7. Allard C, Desgagné V, Patenaude J, et al (2015) Mendelian randomization supports causality between maternal hyperglycemia and epigenetic regulation of leptin gene in newborns. Epigenetics 10(4):342–351. https://doi.org/10.1080/15592294.2015.1029700

8. Taschereau A, Doyon M, Arguin M, et al (2025) Cohort profile: the Genetics of Glucose regulation in Gestation and Growth (Gen3G) - a prospective prebirth cohort of mother-child pairs in Sherbrooke, Canada, 3-year and 5-year follow-up visits. BMJ Open 15(3):e093434. https://doi.org/10.1136/bmjopen-2024-093434

9. HAPO Study Cooperative Research Group, Metzger BE, Lowe LP, et al (2008) Hyperglycemia and adverse pregnancy outcomes. N Engl J Med 358(19):1991–2002. https://doi.org/10.1056/NEJMoa0707943

10. Group HSCR (2002) The Hyperglycemia and Adverse Pregnancy Outcome (HAPO) Study. International Journal of Gynecology & Obstetrics 78(1):69–77. https://doi.org/10.1016/S0020-7292(02)00092-9

11. Freathy RM, Hayes MG, Urbanek M, et al (2010) Hyperglycemia and Adverse Pregnancy Outcome (HAPO) Study: Common Genetic Variants in GCK and TCF7L2 Are Associated With Fasting and Postchallenge Glucose Levels in Pregnancy and With the New Consensus Definition of Gestational Diabetes Mellitus From the International Association of Diabetes and Pregnancy Study Groups. Diabetes 59(10):2682–2689. https://doi.org/10.2337/db10-0177

12. Laurie CC, Doheny KF, Mirel DB, et al (2010) Quality control and quality assurance in genotypic data for genome‐wide association studies. Genetic Epidemiology 34(6):591–602. https://doi.org/10.1002/gepi.20516

13. Urbanek M, Hayes MG, Armstrong LL, et al (2013) The chromosome 3q25 genomic region is associated with measures of adiposity in newborns in a multi-ethnic genome-wide association study. Hum Mol Genet 22(17):3583–3596. https://doi.org/10.1093/hmg/ddt168

14. Hayes MG, Urbanek M, Hivert M-F, et al (2013) Identification of *HKDC1* and *BACE2* as Genes Influencing Glycemic Traits During Pregnancy Through Genome-Wide Association Studies. Diabetes 62(9):3282–3291. https://doi.org/10.2337/db12-1692

15. Powe CE, Udler MS, Hsu S, et al (2020) Genetic Loci and Physiologic Pathways Involved in Gestational Diabetes Mellitus Implicated Through Clustering. Diabetes 70(1):268–281. https://doi.org/10.2337/db20-0772

16. Kuang A, Hayes MG, Hivert M-F, Balasubramanian R, Lowe WL, Scholtens DM (2022) Network Approaches to Integrate Analyses of Genetics and Metabolomics Data with Applications to Fetal Programming Studies. Metabolites 12(6):512. https://doi.org/10.3390/metabo12060512

17. Keikkala E, Mustaniemi S, Koivunen S, et al (2020) Cohort Profile: The Finnish Gestational Diabetes (FinnGeDi) Study. Int J Epidemiol 49(3):762–763g. https://doi.org/10.1093/ije/dyaa039

18. Mustaniemi S, Vääräsmäki M, Eriksson JG, et al (2018) Polycystic ovary syndrome and risk factors for gestational diabetes. Endocr Connect 7(7):859–869. https://doi.org/10.1530/EC-18-0076
